# Effect of the COVID-19 Pandemic on Global Interest in Plastic Surgery

**DOI:** 10.1016/j.jpra.2023.05.002

**Published:** 2023-05-21

**Authors:** Melinda Lem, Joshua KyungHo Kim, Jason T. Pham, Cathy J. Tang

**Affiliations:** a Department of Plastic Surgery, University of California Irvine Medical Center; b School of Medicine, Duke University

**Keywords:** Aesthetic Surgery, Cosmetic Plastic Surgery, International Plastic Surgery, Facial plastic surgery, Nonsurgical plastic surgery, Google Trends

## Abstract

**Introduction:**

Due to the SARS-CoV-2 (COVID-19) pandemic, many elective surgeries were canceled, including most aesthetic plastic surgery procedures. Although studies have shown COVID-19’s effect on plastic surgery in the United States, no study to date has examined the international interest in plastic surgery procedures after the start of the COVID-19 pandemic. Thus, we sought to find this effect using the Google Trends tool.

**Material and Methods:**

The most common cosmetic procedures and top countries with the highest plastic surgery volume were selected from the International Society of Plastic Surgeons report and used as the search terms for Google Trends. Weekly search data from each procedure and country were collected from March 18, 2018 to March 13, 2022, split into 2 periods according to the start of the US COVID-19 lockdown, and compared.

**Results:**

Among the countries, the United States had the most plastic surgery interest after the COVID-19 pandemic, with India and Mexico closely following. On the other hand, Russia and Japan had the fewest changes in procedure interest. Regarding specific procedures, interest in breast augmentation, forehead lift, injectable filler, laser hair removal, liposuction, microdermabrasion, and rhytidectomy increased in all countries after the COVID-19 pandemic.

**Conclusions:**

After COVID-19, there has been increasing interest in almost all plastic surgery procedures globally, especially nonsurgical procedures and facial plastic surgery, with the greatest increases in the United States, India, and Mexico. These results can help inform plastic surgeons which procedures to focus on and which devices or technologies to invest in that are specific to their country.

## Introduction

In March 2020, the pandemic of SARS-CoV-2 (COVID-19) incited a cascade of effects globally that have not been seen in a century. These effects included supply chain problems, economic upheaval, public event cancelations, and a global health crisis.^1^^,^^2^ Because of the lack of hospital beds, scarcity of personal protective equipment, and goal to prevent COVID-19 exposure to other patients, many countries restricted the number of elective surgeries during the peak of COVID-19. It is estimated that about 28 million elective surgeries were postponed or canceled worldwide.^3^

From 2019 to 2020, there was a 10.9% decrease in aesthetic surgical procedures internationally, which was due to many plastic and reconstructive surgery procedures being considered elective.^4^, ^5^, ^6^, ^7^ Consistent with this decrease, public interest in plastic surgery also declined during the early stages of the pandemic in the United States.^8^^,^^9^

However, multiple studies have demonstrated that interest in plastic surgery is quickly rebounding and exceeding the pre–COVID-19 interest level in the United States.^8^, ^9^, ^10^ Potential reasons for this include more time at home to recover from a procedure, the effect of video conferencing, and the increased use of different video social media platforms such as TikTok.^11^ These studies used online data and “infoveillance,” such as Twitter hashtags and Google search trends, to track interest levels before surgical volume data became available and reported the results. In particular, the Google Trends tool provides accessible and real-time search data from consumers’ Google search inputs, with daily or weekly resolution, making it a dynamic tool, whereas other data collection methods, including surveys or recordkeeping, may require more effort and are time-consuming.^12^ This was evident during the pandemic, where it was heavily used to track quickly changing information dissemination, consumer interests, and predict COVID-19 rates in geographic regions.^13^^,^^14^ Although the Google Trends tool does not give exact data on and may not always translate to surgical volume, it can be used to determine which procedures are growing in popularity over a specific time period. Overall, this effective tool has the ability to analyze dynamic topics in society by tracking live search query information that indicates quickly changing interests, whereas more traditional datasets collect end-point information as a result of these changing interests, and periodically at the end of a cycle.

Although studies have shown how COVID-19 has affected plastic surgery trends in the United States, no study to date has examined the changing international trends in plastic surgery. Therefore, we sought to find this effect using a useful and real-time tool, which may help inform plastic surgeons as to which procedures to focus on and which devices or technologies to invest in as patient interests change that are specific to their country.

## Materials and Methods

### Data Collection

Data were captured over a 4-year period, from March 18, 2018 to March 13, 2022 and was split into 2 periods, with 2 years either preceding or following the pandemic start date according to the start of the US COVID-19 lockdown.^15^ March 18, 2018 to March 14, 2020 represented the preceding period, and March 15, 2020 to March 19, 2022 defined the period that was affected by the lockdown. Within this time range, relative search interest was recorded on a weekly basis, giving the data high granularity. Relative search interest is a representation of how popular a search term is, and it is calculated by normalizing the search data that Google Trends collects. The search interest for a particular term is divided by the total number of searches conducted within that same geographic area and timeframe. The resulting quotient is scaled from 0 to 100 on the basis of all searches for all topics. Consequently, if 2 terms have the same search interest, but originate from a different region or time frame, they do not necessarily have an identical search volume. Relative search interest best illustrates how search interest trends or has changed over time, not the search volume data or prevalence.^12^ In obtaining these data, no information about the identity of internet users is provided or retained.

Relative search interest for keywords relating the highest volume surgical and nonsurgical procedures from the most recent International Society of Aesthetic Plastic Surgeons (ISAPS) annual report and Google Trends (Google Inc.) were queried. The most common procedures were selected from the ISAPS report, resulting in a total of 21 procedures. Separate regional searches were conducted in the 10 countries with the most plastic surgery cases, as reported by ISAPS (Table 1). These countries were the United States, Brazil, Germany, Japan, Turkey, Mexico, Argentina, Italy, Russia, and India. Each search was restricted to “Web” results from “All categories,” with low search volume results excluded.

**Table 1:** Top 10 countries with highest plastic surgery procedural volume in 2020 (International Society of Aesthetic Plastic Surgery).

Country	Total procedures	Percentage of total (%)
United States of America	4,667,931	19.0%
Brazil	1,929,359	7.9%
Germany	1,157,738	4.7%
Japan	1,058,198	4.3%
Turkey	945,477	3.9%
Mexico	860,718	3.5%
Argentina	850,840	3.5%
Italy	830,868	3.4%
Russia	621,600	2.5%
India	524,064	2.1%
**Worldwide**	**24,529,875**	**100%**

On the Google Trends tool, one would typically obtain data by looking at search terms, which are the exact words that a person would input into the Google search website. However, to account for language differences and regional colloquialisms, procedures were searched by topic codes rather than exact search terms (Table 2). Topic codes are a string of characters or symbols that Google Trends interprets as representing a collection of phrases or terms that are closely related. In this case, topic codes represent phrases related to the procedure of interest, as well as their equivalents in other languages. When Google Trends is queried with topic codes, the search interest for all terms it represents is aggregated and returned. Surgical and nonsurgical procedures that did not have corresponding topic codes were excluded from the analysis.

**Table 2:** Plastic Surgery Procedure and Associated Google Trends Search Topic Link.

Procedure	Classification	Code
Abdominoplasty	Surgical	/m/01_mbc
Belt lipectomy	Surgical	/m/0cqty9
Blepharoplasty	Surgical	/m/06vthy
Brachioplasty	Surgical	/m/03cwk5l
Breast Augmentation	Surgical	/m/0g54q10
Breast Reduction	Surgical	/m/02z0g2
Buttock Augmentation	Surgical	/m/076ccs
Chemical peel	Nonsurgical	/m/0fd348
Dermaplaning	Nonsurgical	/g/11j2fv6dkb
Forehead Lift	Surgical	/m/02q50zy
Gynecomastia surgery	Surgical	/m/07cszr
Injectable Filler	Nonsurgical	/m/0wx_40 m
Labiaplasty	Surgical	/m/07q9fn
Laser Hair Removal	Nonsurgical	/m/02 × 11_
Liposuction	Surgical	/m/0bk99
Mastopexy	Surgical	/m/0cvp1f
Microdermabrasion	Nonsurgical	/g/187cczy1
Microneedling	Nonsurgical	/g/11fsrbqtzk
Otoplasty	Surgical	/m/04n3km
Rhinoplasty	Surgical	/m/01pppk
Rhytidectomy	Surgical	/m/051__c

### Data Analysis

Equal variance and normality were not guaranteed within the data, as determined by Levene and Shapiro-Wilk tests, so a 2-tailed Mann-Whitney U test was applied to determine whether there was a difference in means between the 2 periods for each procedure and country pairing, with significance set at p < 0.05. Effect size was calculated, with values between 0.3 and 0.5 being deemed moderate and values exceed 0.5 being regarded as large. Analysis was conducted in R version 4.2.0 (The R Foundation for Statistical Computing) in RSTudio using the rstatix and stats packages.

## Results

### Overall Trends

Among the countries, the United States had the highest number of significant plastic surgery interest trends (n = 18) with India and Mexico tied for second (n = 14). Conversely, Russia (n = 10) and Japan (n = 7) had the fewest number of significant changes in procedure search interest since before the COVID-19 pandemic.

Among the procedure types, 7 had notable changes in search interest in all countries—breast augmentation, forehead lift, use of injectable filler, laser hair removal, liposuction, microdermabrasion, and rhytidectomy. Searches for 6 procedures—breast reduction, chemical peel, labiaplasty, mastopexy, otoplasty, and rhinoplasty—exhibited changes in 9 of 10 countries. Searches for abdominoplasty, blepharoplasty, and gynecomastia surgery showed changes in 8 countries.

The largest effect sizes, denoting the magnitude of change in search interest, were recorded predominantly in Brazil, Germany, and the United States (Table 3). Brazilian searches for dermaplaning (r = 0.83, p < 0.001) showed the greatest change between before and after the pandemic period. Other searches in Brazil were notable, such as rhytidectomy (r = 0.78, p < 0.001), mastopexy (r = 0.71, p < 0.001), and liposuction (r = 0.68, p < 0.001). Searches for forehead lifts in Germany (r = 0.79, p < 0.001) also displayed notable increases following the pandemic start date.

**Table 3:** Plastic surgery procedures that have both a statistically significant trend and large effect magnitude.

Procedure	Country	P Value	Effect Size
Abdominoplasty	US	3.01E-17	0.5843082606
Blepharoplasty	BR	1.70E-15	0.5508109141
Blepharoplasty	US	3.30E-17	0.5835663233
Breast Augmentation	RU	7.31E-17	- 0.5771057359
Breast Augmentation	TR	3.15E-13	- 0.5041897242
Breast Reduction	US	3.62E-21	0.6532663878
Buttock Augmentation	DE	9.18E-23	0.6793817083
Chemical peel	IN	2.90E-21	0.6548873207
Chemical peel	JP	2.78E-17	0.5849503362
Chemical peel	MX	8.92E-14	0.5158219371
Dermaplaning	BR	2.17E-33	0.832958753
Forehead Lift	BR	1.22E-20	0.6444020112
Forehead Lift	DE	6.59E-30	0.785886477
Forehead Lift	US	8.48E-28	0.7559781782
Hair removal	IN	1.78E-30	0.7937520175
Hair removal	US	5.59E-21	0.6501172135
Injectable Filler	AR	4.55E-13	0.5007399685
Injectable Filler	IT	4.26E-13	0.5013581126
Injectable Filler	US	2.70E-23	0.6878529729
Labiaplasty	BR	1.08E-15	0.5546442977
Laser Hair Removal	BR	4.97E-15	0.541532095
Laser Hair Removal	IN	5.44E-18	0.5979830634
Laser Hair Removal	US	6.28E-17	0.5783407544
Liposuction	BR	8.69E-23	0.6797625415
Liposuction	MX	4.91E-20	0.6341047664
Liposuction	US	1.98E-20	0.6408483529
Mastopexy	BR	9.83E-25	0.7103276741
Microdermabrasion	MX	6.50E-20	- 0.6320042855
Otoplasty	US	5.64E-17	0.5792127417
Rhinoplasty	IN	5.22E-22	0.6671550825
Rhinoplasty	US	1.92E-21	0.6578451333
Rhytidectomy	AR	9.28E-15	0.5360669838
Rhytidectomy	BR	2.17E-29	0.7786507294
Rhytidectomy	MX	4.07E-13	0.5017863233
Rhytidectomy	RU	2.29E-16	0.5676855345
Rhytidectomy	TR	1.55E-14	0.5315492437
Rhytidectomy	US	7.27E-17	0.5771511772

AR = Argentina, BR = Brazil, DE = Germany, IN = India, IT = Italy, JP = Japan, MX = Mexico, RU = Russia, TR = Turkey, US = United States of America

### Interest Trends by Surgical Procedure

All data reported below have a p value of <0.001. Out of all 10 countries, only the United States had a large increase in interest in abdominoplasty (r = 0.58), whereas India showed a moderate decrease in interest (r = 0.46). Interest in blepharoplasty was significantly increased in the United States and Brazil (r = 0.55, 0.58). Interest in breast augmentation procedures greatly decreased in Russia and Turkey (r = 0.57, 0.50) and moderately decreased in Germany, India, and Japan (r = 0.40, 0.34, 0.33). Interest in breast reduction surgery greatly increased in the United States (r = 0.65) and moderately decreased in Turkey (r = 0.31). Interest in forehead lift, also known as brow lift, was increased in Brazil, Germany, and the United States (r = 0.64, 0.78, 0.75). Only Brazil showed an increase in interest in labiaplasty (r = 0.55). Increases in liposuction interest were observed in Brazil, Mexico, and the United States (r = 0.67, 0.63, 0.64). Only Brazil had an increase in mastopexy interest (r = 0.71). Only the United States had an increase in interest in otoplasty (r = 0.57). Both India and the United States had a large increase in rhinoplasty interest (r = 0.66, 0.65). Six countries, Argentina, Brazil, Mexico, Russia, Turkey, and the United States, had a large increase in rhytidectomy interest (r = 0.53, 0.77, 0.50, 0.56, 0.53, 0.57).

### Interest Trends by Nonsurgical Procedure

All data reported below have a p-value of <0.001. Injectable filler interest was largely increased in Argentina, Italy, and the United States (r = 0.50, 0.50, 0.68). Interest in chemical peel increased in India, Japan, and Mexico (r = 0.65, 0.58, 0.51) and moderately decreased in Russia (r = 0.37). Only Brazil had a large increased interest in dermaplaning (r = 0.83). Interest in laser hair removal was increased in Brazil, India, and the United States and decreased in Japan (r = 0.54, 0.59, 0.57, 0.44). Interest in microdermabrasion was decreased moderately in Germany, India, and Turkey (r = 0.41, 0.32, 0.32) and greatly in Mexico (r = 0.63).

### Interest Trends by Country

All data reported below have a p value of <0.001. In Argentina, the top increasing search trends were rhytidectomy, injectable filler, and mastopexy (r = 0.53, 0.50, 0.36). In Brazil, increasing search trends that had a large effect magnitude were dermaplaning, rhytidectomy, mastopexy, liposuction, forehead lift, labiaplasty, blepharoplasty, and laser hair removal (r = 0.83, 0.77, 0.71, 0.67, 0.64, 0.55, 0.55, 0.54). In Germany, the top increasing search trends were forehead lift, buttock augmentation, and rhytidectomy (r = 0.78, 0.67, 0.47). Both microdermabrasion and breast augmentation interest decreased moderately in Germany (r = 0.41, 0.40). Interest in rhinoplasty, chemical peel, and laser hair removal were largely increased in India (r = 0.66, 0.65, 0.59). Additionally, interest in abdominoplasty, breast augmentation, and microdermabrasion were moderately decreased in India (r = 0.46, 0.34, 0.32). There was increased interest in injectable filler and chemical peel in Italy (r = 0.50, 0.37). In Japan, interest in chemical peels greatly increased, whereas interest in laser hair removal and breast augmentation moderately decreased (r = 0.58, 0.44, 0.33). In Mexico, interest in liposuction, chemical peel, and rhytidectomy increased, whereas interest in microdermabrasion decreased (r = 0.63, 0.51, 0.50, 0.63). Russia showed increased interest in rhytidectomy, labiaplasty, and abdominoplasty and decreased interest in breast augmentation and chemical peels (r = 0.56, 0.40, 0.35, 0.57, 0.37). In Turkey, interest in rhytidectomy, labiaplasty, and rhinoplasty increased, whereas interest in breast augmentation, microdermabrasion, and breast reduction decreased (r = 0.53, 0.48, 0.42, 0.50, 0.32, 0.31). In the United States, interest in 15 procedures showed a large to moderate increase without any decreased interest in any procedure. The top 5 procedures with increased interest were forehead lift, injectable filler, rhinoplasty, breast reduction, and liposuction (r = 0.0.75, 0.68, 0.65, 0.65, 0.64).

## Discussion

In 2020, a total of 24,529,875 plastic surgery procedures were performed worldwide, including breast augmentation, injectable fillers, upper arm lift, skin laser rejuvenation, rhinoplasty, and hair removal.^7^ However, this was a 1.8% decrease in total plastic surgery procedures from 2019, mainly due to the 10.9% decrease in surgical procedures. This can be explained by the cancelation of elective surgeries during the COVID-19 pandemic in 2020.^6^ With the distribution of vaccinations worldwide and the decrease in the number of COVID-19 cases, aesthetic procedures are returning to many hospitals, and patients are starting to seek out these procedures again.

**Figure 1:**
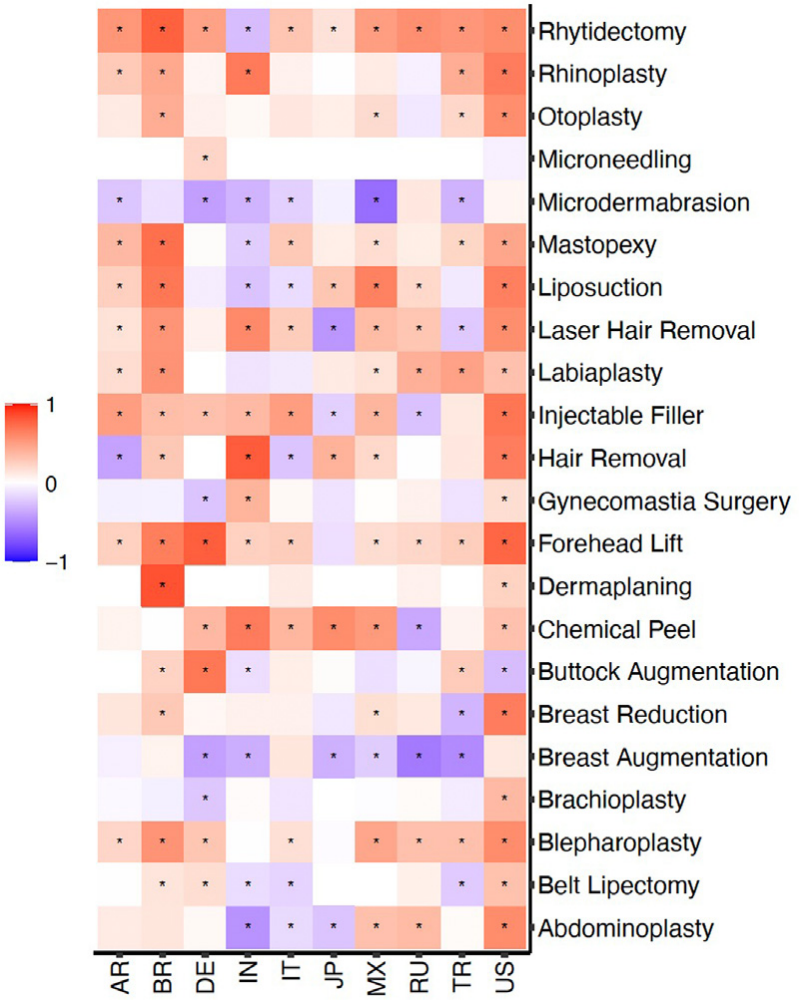
Heat map of the effect size of plastic surgery interest trends by country. AR = Argentina, BR = Brazil, DE = Germany, IN = India, IT = Italy, JP = Japan, MX = Mexico, RU = Russia, TR = Turkey, US = United States of America. * For visual purposes, interest trends that decreased were made artificially negative because all effect size magnitudes were calculated as positive.

In the field of plastic surgery, the International Society of Aesthetic Plastic Surgery, The Aesthetic Society, and American Society of Plastic Surgery provide beneficial data on surgical volume. However, these data are approximately 1 to 2 years delayed, which may be too late for surgeons and surgical centers to plan accordingly for patients’ demand and quickly changing interests. By using the Google Trends tool, we investigated whether there were changes in global interest in the most common surgical and nonsurgical plastic surgery procedures. Consistent with this aim, our results showed that every single nonsurgical or surgical procedure has grown in interest and popularity in at least 1 country after the COVID-19 pandemic (Figure 1).

Rhytidectomies were the most common trending procedure after COVID-19, with interest of moderate to large effect sizes in 7 countries. Interestingly, rhytidectomy was ranked ninth in all worldwide surgical plastic surgery procedures in 2020.^7^ In contrast, breast augmentation, which is ranked first as the most popular plastic surgery worldwide, showed the greatest decline in interest in all countries. The procedures that did not show as much change in interest were microneedling, dermaplaning, brachioplasty, and gynecomastia surgery. The types of procedures that showed large interest changes are notable as well. Specifically, global interest in facial aesthetic procedures increased more than other body procedures, and nonsurgical procedures increased as well. This is consistent with the literature, which showed an increase in facial and above-the-shoulder plastic surgery in the United States after the start of the pandemic, possibly due to the increase in social media use and video conferencing (Aesthetics Society 2021).^11^^,^^16^

By country, Brazil had the most statistically significant increases in procedure interest, and the United States had the largest increases in the most plastic surgery procedures before and after COVID-19. Compared with the ISAPS 2020 ranking of countries with the most procedures, our ranking of countries by most increased interest trends differs (Table 1). After Brazil and the United States, India had the most change in procedure interest, followed by Mexico and Germany. On the ISAPS 2020 list, India was ranked tenth, Mexico sixth, and Germany third. These search results and differences may reveal that there are new rapidly growing markets of aesthetic surgery being formed after COVID-19. In stark contrast, Italy did not have any moderate or large effect size increases in surgical procedure interest. Italy did show an increased interest in 2 nonsurgical procedures, injectable filler and chemical peel, but this stood out from the rest of the countries. This may be explained by the differences between countries from the effect of COVID-19 and the country's response. Italy had an extremely devastating journey with COVID-19, with daily death rates at 800 for months in 2020.^17^ This possibly affected the general public's interest and priorities in nonessential medical procedures, such as cosmetic surgery, which resulted in fewer searches and interest in cosmetic surgery.

Our study's limitations include the usability and applicability of Google Trend data themselves. The data only show interest and do not show actual procedure volume or completion. Additionally, since the data are indexed with a maximum value of 100, they cannot be used for comparisons. For example, 95 interest data from Brazil do not convey any meaning and do not mean the same as 95 interest data from the United States because the data do not note volume and cannot be compared outside their timeframe or region. A further limitation of the study is that Google Trends only includes people who use the Google website to search procedures. This precludes those who do not have access to technology or the internet, do not search with Google, or are interested in the listed procedure but do not search for it. Google Trends topics also use artificial technology to group related terms together, but because the authors are unable to view the included terms, only related topics, we can only infer that the search topics are accurate. Several popular aesthetic procedures were excluded from our analysis due to the lack of search topics for these procedures in the Google Trends tool; therefore, we were not able to control for language and colloquial terms. This included fat grafting, neck lift, facial bone contouring, thigh lift, Botox, and nonsurgical fat reduction.

Although there have been studies that analyzed changing plastic surgery interest within the United States, no study to our knowledge has analyzed international trends.^8^^,^^11^^,^^18^ Because it has been shown that Google Trends data are highly correlated with actual surgical volume, practicing plastic surgeons can use Google Trends data to anticipate changes in procedure interest and volume of cases regardless of their region.^19^ After COVID-19, interest in almost all plastic surgery procedures has increased globally, especially nonsurgical procedures and facial plastic surgery. Additionally, it was shown that the United States, India, and Mexico had the largest increases in plastic surgery interest since the COVID-19 pandemic. This study can be used to guide plastic surgeons to anticipate their country's upcoming plastic surgery trends and invest in the technology to meet patients’ needs.

## Conflict of Interest Statement

None.
